# Supplementary material for: A Regulatory SRNA Rli43 Is Involved in the Modulation of Biofilm Formation and Virulence in Listeria monocytogenes
Source: Pathogens. 2022 Sep 30;11(10):1137. doi: 10.3390/pathogens11101137 (PMC9606912; doi:10.3390/pathogens11101137)
Supplement: Supplementary file 1 [file pathogens-11-01137-s001.zip › pathogens-1855196-supplementary.pdf]

Supplementary Information

Table S1 Determination of LD_{50} of LM EGD-e, LM-*Δrli43* and LM-*Δrli43-rli43* in mice

**Group**	**LM EGD-e**			**LM-*Δrli43***			**LM-*Δrli43-rli43***		
	**Dose/cfu**	**Mortality**	**LD_{50}**	**Dose/cfu**	**Mortality**	**LD_{50}**	**Dose/cfu**	**Mortality**	**LD_{50}**
1	1.68×10^{6}	(10/10)		2.1×10^{9}	100 (10/10)		2.68×10^{6}	100 (10/10)	
2	8.40×10^{5}	(9/10)	$10^{5.56}$	2.1×10^{8}	80 (8/10)	$10^{7.32}$	1.34×10^{6}	80 (8/10)	$10^{5.76}$
3	4.20×10^{5}	(6/10)		2.1×10^{7}	50 (5/10)		6.20×10^{5}	70 (7/10)	
4	2.10×10^{5}	(2/10)		2.1×10^{6}	20 (2/10)		3.10×10^{5}	20 (2/10)	
5	1.05×10^{5}	(0/10)		2.1×10^{5}	0 (0/10)		1.52×10^{5}	0 (0/10)	

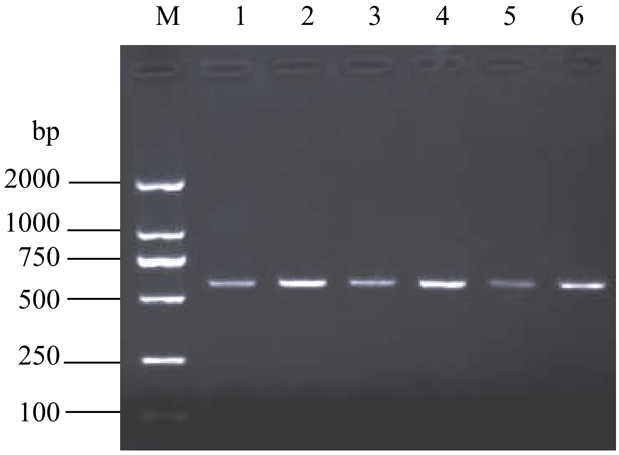

Figure S1 Amplification of LM *rli43* gene by RT-PCR. M: DNA marker DL-2000 (2000, 1000, 750, 500, 250, 100 bp); 1-6: Amplified products of *rli43* gene by RT-PCR.

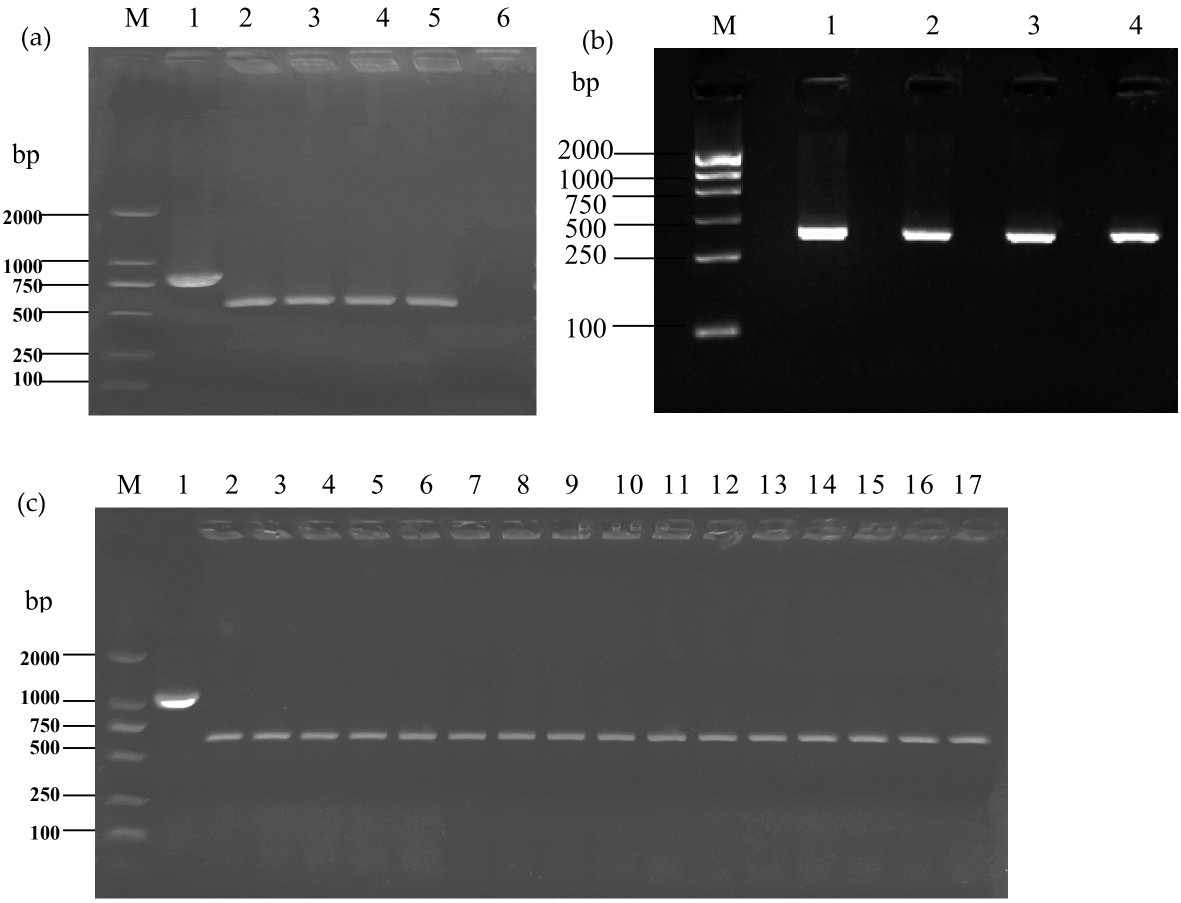

Figure S2 Construction of LM-*Δrli43* deletion and complementary strains. (a) Screening and identification of recombinant LM-*△rli43* by PCR. M: DNA marker DL-2000 (2000, 1000, 750, 500, 250, 100 bp); 1: LM EGD-e strain (positive control); 2-5: Amplified products of recombinant LM-*△rli43* by PCR, 6: Negative control. (b) Screening and verification of complement strain LM-*△rli43-rli43* by PCR. M: DNA marker DL-2000 (2000, 1000, 750, 500, 250, 100 bp); 1-4: Amplified products of complement strain LM-*△rli43-rli43* by PCR. (c) Analysis of genetic stability of LM-*△rli43* by PCR. M: DNA marker DL-2000 (2000, 1000, 750, 500, 250, 100 bp); 1: LM EGD-e strain (positive control); 2-17: PCR Identification of LM-*△rli43* of 1, 2, 4, 5, 7, 10, 12, 13, 16, 19, 20, 22, 25, 26, 27, 30 generation.

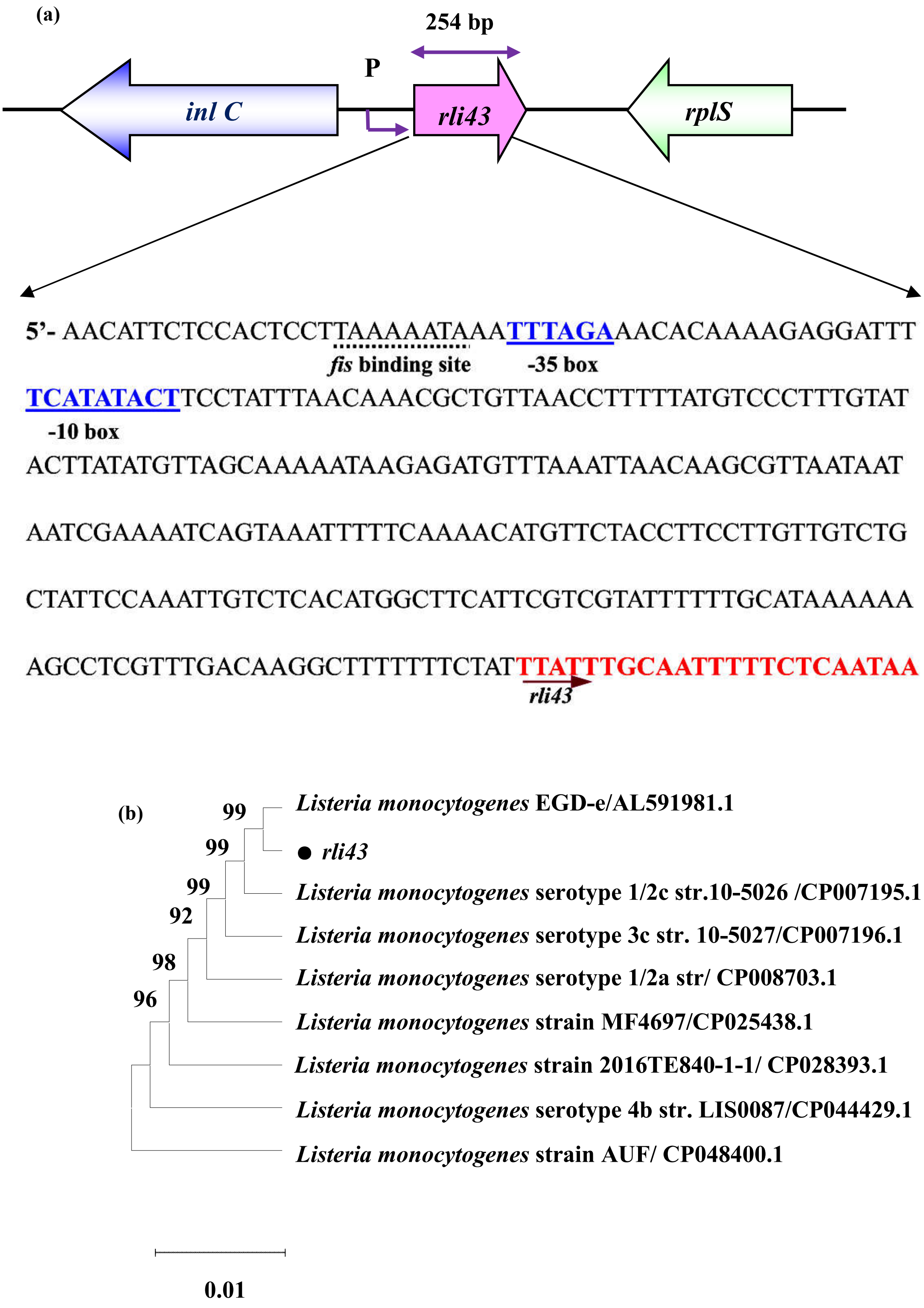

Figure S3 Molecular characterization of *rli43* gene and its phylogenitic analysis. (a) Genetic location of *rli43* gene in the genome of LM EGD-e; (b) Phylogenitic analysis of *rli43* gene of different serotypes of LM.

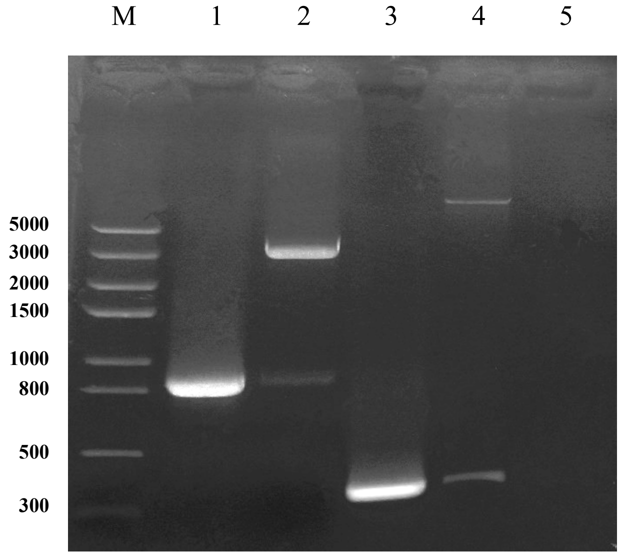

Figure S4 Screening and identification of pMR-LacZ-*htrA* and pUT18C-*rli43* using PCR and restriction enzyme digestion. M: DNA marker DL-5000 (5000, 3000, 2000, 1500, 1000, 800, 500, 300 bp); 1: Identification of pUT18C-*rli43* by PCR; 2: pUT18C-*rli43* digested by restriction enzyme; 3: Verification of pMR-LacZ-*htrA* by PCR; 4: pMR-LacZ-*htrA* digested by restriction enzyme; 5: Negative control.
